# Evaluation of Diagnostic Accuracy, Feasibility and Client Preference for Rapid Oral Fluid-Based Diagnosis of HIV Infection in Rural India

**DOI:** 10.1371/journal.pone.0000367

**Published:** 2007-04-11

**Authors:** Nitika Pant Pai, Rajnish Joshi, Sandeep Dogra, Bharati Taksande, S.P. Kalantri, Madhukar Pai, Pratibha Narang, Jacqueline P. Tulsky, Arthur L. Reingold

**Affiliations:** 1 Immunodeficiency Service, Montreal Chest Institute, McGill University Health Center, Montreal, Canada; 2 Mahatma Gandhi Institute of Medical Sciences, Sevagram, Maharashtra, India; 3 Acharya Shri Chander College of Medical Sciences, Jammu, India; 4 Department of Epidemiology, Biostatistics and Occupational Health, McGill University, Montreal, Canada; 5 Department of Internal Medicine, University of California at San Francisco, San Francisco, California, United States of America; 6 Division of Epidemiology, University of California at Berkeley, Berkeley, California, United States of America; Institute of Human Virology, United States of America

## Abstract

**Background:**

Oral fluid-based rapid tests are promising for improving HIV diagnosis and screening. However, recent reports from the United States of false-positive results with the oral OraQuick® ADVANCE HIV1/2 test have raised concerns about their performance in routine practice. We report a field evaluation of the diagnostic accuracy, client preference, and feasibility for the oral fluid-based OraQuick® Rapid HIV1/2 test in a rural hospital in India.

**Methodology/Principal Findings:**

A cross-sectional, hospital-based study was conducted in 450 consenting participants with suspected HIV infection in rural India. The objectives were to evaluate performance, client preference and feasibility of the OraQuick® Rapid HIV-1/2 test**s**. Two Oraquick® Rapid HIV1/2 tests (oral fluid and finger stick) were administered in parallel with confirmatory ELISA/Western Blot (reference standard). Pre- and post-test counseling and face to face interviews were conducted to determine client preference. Of the 450 participants, 146 were deemed to be HIV sero-positive using the reference standard (seropositivity rate of 32% (95% confidence interval [CI] 28%, 37%)). The OraQuick test on oral fluid specimens had better performance with a sensitivity of 100% (95% CI 98, 100) and a specificity of 100% (95% CI 99, 100), as compared to the OraQuick test on finger stick specimens with a sensitivity of 100% (95% CI 98, 100), and a specificity of 99.7% (95% CI 98.4, 99.9). The OraQuick oral fluid-based test was preferred by 87% of the participants for first time testing and 60% of the participants for repeat testing.

**Conclusion/Significance:**

In a rural Indian hospital setting, the OraQuick® Rapid- HIV1/2 test was found to be highly accurate. The oral fluid-based test performed marginally better than the finger stick test. The oral OraQuick test was highly preferred by participants. In the context of global efforts to scale-up HIV testing, our data suggest that oral fluid-based rapid HIV testing may work well in rural, resource-limited settings.

## Introduction

Rapid point-of-care HIV testing is a very important component of HIV control initiatives and programs. In particular, non-invasive, simple, accurate oral fluid-based rapid tests have the potential to make a big impact on HIV screening programs, especially in areas where laboratory infrastructure is poor or unavailable. Oral fluid-based testing also opens the possibility of home-based HIV testing. The OraQuick ADVANCE® HIV1/2 test (OraSure Technologies Inc, Philadelphia, USA) is the first and only rapid test to be approved by the US Food and Drug Administration (FDA) for use in oral fluid, finger stick, whole blood and plasma specimens. While several studies have shown this test to be accurate in many settings,[1], [2] in December 2005, unusually high rates of false-positive results with the oral fluid-based OraQuick® ADVANCE HIV1/2 test were reported in select cities in the United States. (notably, San Francisco and New York City).[3], [4] This raised concerns about the overall performance of oral fluid testing in general, and led to speculations that oral fluid tests perform worse than blood-based rapid HIV tests.[Fig pone-0000367-g001]

**Figure 1 pone-0000367-g001:**
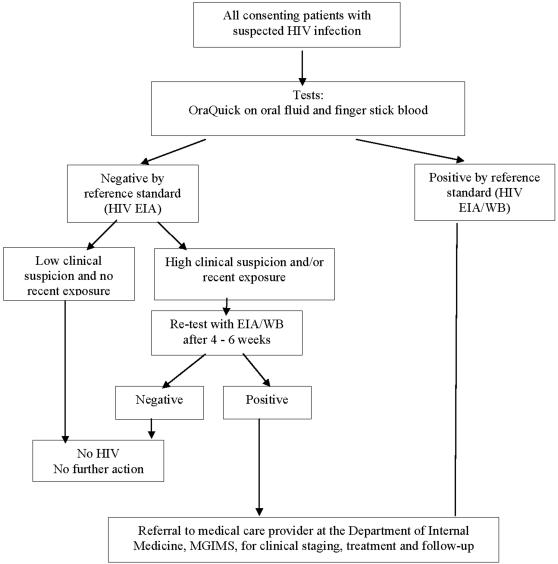
HIV Testing Algorithm

In the case of San Francisco and New York city, it was initially unclear whether factors such as lot variation, product shelf life, collection techniques, storage temperature, and site conditions affected the accuracy of the oral OraQuick test.[4] Following these reports, the US Centers for Diseases Control and Prevention (CDC) recommended a parallel testing strategy with the use of two OraQuick tests, followed by confirmation of test results with a reference standard.[5] Recently, the CDC conducted an investigation into the cluster of false-positive test results with oral fluid OraQuick test in Minnesota [6]. This investigation failed to identify a cause for the increase in false-positive test results from an isolated cluster.[6] Thus, there is some lingering skepticism regarding the field performance of the oral fluid-based HIV test. In this context, there is a need for real world field studies to evaluate the accuracy and performance characteristics of oral fluid-based rapid HIV testing, especially in resource limited settings where they can contribute the most. We evaluated the diagnostic accuracy of the OraQuick rapid HIV 1/2 test in a hospital setting in rural India.

India has the second largest number of HIV infected people in the world, second only to South Africa.[7] However, it has been reported that HIV prevalence in southern Indian States is on the decline [8] Knowledge of sero-status is the cornerstone of HIV prevention, diagnosis and linkages to care and prevention. Many Indians including rural poor, are unaware of their sero-status. [9] Rapid point-of-care HIV tests can greatly aid in knowing sero-status by providing faster and accurate results in minutes. In India, rapid HIV tests currently marketed are blood based tests that are cumbersome, require trained laboratory technicians, and test results are often not available at the point-of-care. Moreover, in rural areas resources are limited, laboratory technicians frequently unavailable, phlebotomy is difficult and culturally less preferred. Therefore, follow up on HIV test results can prove to be very challenging. In such settings, oral fluid-based rapid tests provide a convenient and feasible alternative to blood-based testing.[2] Although oral fluid-based rapid testing has several attractive features, few studies have evaluated the latest versions of the OraQuick Rapid HIV1/2 tests in rural India. We report the results of a cross-sectional hospital-based study conducted in Maharashtra state in India.

Our study objectives were: 1) to determine the diagnostic accuracy of the OraQuick Rapid HIV-1/2 tests (performed independently on both oral mucosal transudate (OMT) and finger stick blood specimens) in a parallel testing strategy, 2) to determine client preference for oral fluid-based testing in a rural hospital setting, and 3) to evaluate feasibility of conducting oral fluid tests in a rural resource constrained setting.

## Materials and Methods

### Study setting

Our study was conducted at the Mahatma Gandhi Institute of Medical Sciences (MGIMS), a rural teaching hospital in Sevagram, Central India. This busy 650-bed tertiary hospital has over 350,000 patient visits each year. Our study was conducted between October 2004 and December 2005. No community-based HIV prevalence data exist for Sevagram. The study was approved by ethics committees at the University of California, Berkeley, and the Mahatma Gandhi Institute of Medical Sciences, Sevagram, India. A written informed consent was obtained from all participants, and all HIV tests were done after appropriate counseling.

### Study participants: inclusion and exclusion criteria

Participants for this study were recruited from both inpatient and outpatient facilities of the departments of internal medicine, and dermatology (which also provides care for patients with sexually transmitted infections). A convenience-based sampling method was employed for recruiting participants. Consenting participants were interviewed and provided pre-test and post-test counseling as per the guidelines of the National AIDS Control Organization (NACO), India.

Participants were administered index and reference standard tests concurrently. Data on demographic characteristics and client preference for various HIV testing modalities was also collected, along with data on risk factors for HIV.

Participants were eligible if any of the following criteria were met: a) an adult (>18 years of age); and b) presence of signs and/or symptoms of HIV infection (i.e., unexplained weight loss for more than 6 months; unexplained fever for more than 3 months; chronic cough or weight loss for more than 3 months; loose stools for more than 1 month; generalized lymphadenopathy of non malignant origin); c) or, presence of signs and symptoms of opportunistic infections (i.e., pulmonary or extrapulmonary tuberculosis, candidiasis, *Pneumocystis jiroveci* pneumonia, aseptic meningitis, toxoplasmosis, primary central nervous system lymphoma, diarrhea, or Kaposi's sarcoma); d) or, presence of one or more risk factors for HIV infection, (i.e., concurrent sexually transmitted infection, spouse of a known HIV-positive individual, recent unprotected intercourse with a commercial sex worker, intravenous drug user, recipient of multiple unscreened blood products). Participants were excluded if they were: a) pregnant or breast feeding; or b) had chronic debilitating conditions or mental health disorders that would preclude informed consent.

### HIV test methods and lab procedures

After appropriate pre-test counseling by a trained health worker, all eligible and consenting participants received the following index tests (OraQuick rapid tests performed on oral-fluid as well as finger stick blood specimens) and the reference standard. The reference standard (Table 1) was based on the CDC guidelines for rapid testing. The reference standard used for confirmation of preliminary positives was a combination of repeat enzyme-linked immunoassay (ELISA) and Western Blot. For confirmation of preliminary negatives, one ELISA test alone was used as the reference standard.

**Table 1:** Testing algorithm and reference standard

Scenario	OraQuick oral fluid	OraQuick finger stick		ELISA#1	ELISA#2	Western Blot* [Table-fn nt102]	Tests performed
1	NEG	NEG	then	NEG then STOP	Not done	Not done	Testing stopped with one EIA
2	NEG	NEG	then	POS	NEG	Done	All tests performed
3	POS	POS	then	POS	POS	Done	All tests performed
4	POS	NEG	then	POS	NEG	Done	All tests performed
5	NEG	POS	then	POS	POS	Done	All tests performed

* If Western Blot was indeterminate, ELISA (enzyme-linked immuno-assay) was repeated on stored sera; if repeatedly indeterminate, the sequence was repeated with a fresh blood sample

* Ref: CDC guidelines for rapid testing

Flowchart 1 illustrates the overall testing protocol and algorithm employed. The OraQuick® rapid HIV1/2 tests were applied independently and in parallel, to oral mucosal fluid specimens and to finger stick blood specimens, according to the manufacturer's instructions. Standard criteria recommended by the manufacturer (OraSure Technologies, Inc, PA, USA) were used to classify the results as “negative,” “reactive,” or “invalid.” For confirmatory testing, venipuncture was performed on participants, and blood samples were collected for ELISA [Vironostika® HIV Uniform II Ag/Ab Plus O; Organon Teknika Corp, NC, USA] and Western Blot tests (Qualicode HIV1/2 Kit; Immunetics technologies, Boston, USA].

Testing was conducted in a double-blind manner. Each test negative participant received two Oraquick tests and one ELISA test. Each test positive participant received two Oraquick tests, two ELISA tests, one internal Western Blot and one external Western Blot (Table 1). The external Western Blot test was conducted by an external reference laboratory, as part of quality assurance. The trained health worker who performed OraQuick tests was unaware of the reference standard results. The laboratory technician performing the ELISA and Western Blot assays was blinded to the results of the OraQuick rapid tests. However, both OraQuick tests (oral fluid and finger stick) were performed by the same health worker at the same time, and thus not blinded. After HIV testing, test results were communicated to the patient by a trained health worker. Post-test counseling was conducted per NACO guidelines. [9]

Questionnaires were administered in face to face interviews by trained health workers. After pre-test counseling session, information on demographics, risk factors was obtained from participants. After post-test counseling, information on past and current HIV test experience and preference for current test methods was obtained and recorded. [9]

#### Statistical Analysis

Analysis was conducted using Stata software (version 9.0; Stata Corp, College Station, TX, USA). The main outcomes were diagnostic accuracy measures, estimated using sensitivity, specificity, and predictive values, along with 95% confidence intervals (CI). Concordance between test results were estimated using kappa statistic.

## Results

### Description of the study population

A total of 450 participants were recruited and tested for HIV. The median age of the study participants was 34 years (range 18–88 years). Of the 450 participants, 74% were men. Most patients (65%) were rural laborers and farmers. About 39% of participants presented with signs and symptoms suggestive of HIV infection; 44% had signs and symptoms suggestive of opportunistic or AIDS defining illnesses, and 17% had one or more risk factors for HIV infection. About 9% of the participants had been tested for HIV infection in the past.

### Prevalence of HIV infection using reference standard

Of the 450 participants, 146 were deemed HIV positive using the reference standard of ELISA and Western Blot, yielding a prevalence of 32% (95% CI 28%, 37%). In all cases, ELISA and Western Blot results were 100% concordant.

### Diagnostic accuracy of OraQuick tests

All 450 participants underwent both OraQuick and ELISA/Western Blot tests. Acceptability of the test refers to the proportion of people who agreed to get tested voluntarily of the eligible persons who were offered testing. In our study, the test acceptability was 100% (450/450). For all patients, OraQuick results were obtained between 20–40 minutes, while ELISA and Western Blot results were available within 2 weeks of phlebotomy. No OraQuick test result was reported as indeterminate or invalid.

As shown in Table 2, the OraQuick test performed on oral fluid specimens had a sensitivity of 100% (95% CI 98, 100), and specificity of 100% (95% CI 99, 100). Thus, the oral fluid test had 100% positive and negative predictive values. As shown in Table 3, the OraQuick test performed on finger stick specimens had a sensitivity of 100% (95% CI 98, 100), and a specificity of 99.7 (95% CI 98.4, 99.9). Agreement between the oral and finger stick OraQuick test results was high (kappa = 0.99; 95% CI 0.99, 1.00)).

**Table 2:** Diagnostic accuracy of the OraQuick HIV-1/2 rapid test performed on oral mucosal fluid specimens

	ELISA+Western Blot positive	ELISA+Western Blot negative	Total
Oral fluid test positive	146	0	146
Oral fluid test negative	0	304	304
	146	304	450

Sensitivity = 100% (95% CI 98, 100)

Specificity = 100% (95% CI 99, 100)

Positive predictive value = 100%

Negative predictive value = 100%

Positive likelihood ratio = NA

Negative likelihood ratio = 0

**Table 3:** Diagnostic accuracy of the OraQuick HIV-1/2 rapid test performed on finger stick blood specimens

	ELISA+Western Blot positive	ELISA+Western Blot negative	Total
Finger stick test positive	146	1	147
Finger stick test negative	0	303	304
	146	304	450

Sensitivity = 100% (95% CI 98, 100)

Specificity = 99.7% (95% CI 98.4, 99.9)

Positive predictive value = 98.17%

Negative predictive value = 100%

Positive likelihood ratio = 304

Negative likelihood ratio = 0

Only one participant was found to be finger stick positive, but oral test negative. In this participant, the reference standard tests (ELISA/Western Blot) were negative on first time testing. On retesting at 3 months, both OraQuick tests (i.e. finger stick and oral) and the reference standard tests were negative. Thus, the OraQuick finger stick test yielded one false positive result (false positivity rate of 0.3%).

### Client preference for various HIV testing modalities

Client preference was evaluated using face to face interviews, after testing with oral, finger stick, and venipuncture specimens. When participants were questioned about discomfort during collection of each specimen, a majority (92%) reported no discomfort with Oraquick oral test, followed by 75% for venipuncture-based blood test and lastly, 66% for Oraquick finger stick tests. Discomfort was most often reported with finger stick rapid test method. Further, pain and fear of blood draw during finger stick sample collection was reported by 24% of participants. Participants were questioned after HIV testing regarding test preference. A majority (87%) preferred the oral fluid OraQuick test for first time HIV testing and for re-testing, a majority (60%) chose the OraQuick oral test.

## Discussion

In this study, the first field evaluation of the new and improved OraQuick® rapid HIV-1/2 oral fluid test in rural India, we found that the oral mucosal fluid test was 100% accurate. Accuracy of the finger stick OraQuick test was comparable, although the finger stick test yielded one false positive result (i.e. false positivity rate of 0.3%). A parallel testing strategy reduced the number of false positives. Overall, the concordance between oral fluid and finger stick testing was exceedingly high (kappa 0.99). The oral OraQuick test yielded no false positive results, and all quality assurance procedures were in place during the study period.

Apart from high accuracy, a key finding of our study was the high level of client preference (87%) for the oral fluid-based OraQuick test for initial testing, and 60% for re-testing. Preference for oral HIV tests in previous studies ranged from 33% to 65%. [10], [11], [12] There may be several reasons why our participants preferred the oral fluid-based test: less discomfort, less pain, greater cultural acceptability of giving oral fluid than blood specimens, and novelty of the test. Our data suggest the potential use of OraQuick oral fluid test for scale-up of HIV testing in rural India.

About one-third of the study participants tested positive (seropositivity of 32% (95% CI 28%–37%). These estimates are higher than a previous study in STD clinic attendees, conducted at an urban hospital in India that reported 22% HIV seropositivity.[13] Our study was conducted in a select clinic population in whom testing was indicated using a convenience-based sample. Our study was not designed to estimate the prevalence of HIV infection in the rural population.

How do our data on accuracy compare with previous studies? This is the first report from rural India on high accuracy of the new and improved oral OraQuick rapid HIV1/2 test compared to the finger stick whole blood test. According to the OraSure Technologies (PA, USA), the OraQuick test has a sensitivity of 100% and a specificity of 99.87%. OraQuick tests evaluated in other settings (i.e., labor and delivery settings, emergency departments and correctional facilities) showed sensitivity of 99.3% (95% CI 98.4, 99.7) and specificity of 99.8% (95% CI 99.6, 99.9) for oral fluid-based OraQuick, and sensitivity of 99.6% (95% CI 98.5, 99.9), and specificity of 100% (95% CI 99.7, 100) for whole blood-based OraQuick tests. [1]–[3], [11], [14]–[17]

However, our study results are not consistent with a previous study from India in pregnant women that reported a lower sensitivity for the OraQuick salivary test.[14] Factors that could partially explain the lower accuracy reported by the previous study are: a) use of an earlier version of the OraQuick test, b) use of a single ELISA test of lower diagnostic accuracy as the reference standard. [14]

To summarize, the highly accurate OraQuick oral fluid-based test was preferred by our participants for first time and second time testing over conventional tests. Oral OraQuick test was 100% accurate and performed marginally better than the finger stick OraQuick test. Although one false-positive result was obtained using the finger stick OraQuick test, the overall rate of false-positive OraQuick results was very low, and therefore not consistent with the recent reports from the United States. Furthermore, the OraQuick tests were rapid, easy, feasible, and convenient even in a rural hospital setting in India. These characteristics have the potential to encourage people to present for voluntary HIV testing. Oral fluid-based HIV testing in outreach settings can help expand the existing voluntary testing and counseling program in high prevalence countries such as India. Our study suggests that oral OraQuick test could be used in community-based surveys to estimate the true burden of HIV infection in the country. In the context of global efforts to scale-up HIV testing, our data suggests that oral fluid-based rapid HIV testing may work well in rural resource limited settings, and greatly enhance the control of the HIV epidemic in poor countries. In the view of the high accuracy, feasibility and client preference, it is important to ensure that oral fluid-based rapid tests are made affordable in resource limited countries which need them the most.
